# Role of androgen receptor signaling pathway-related lncRNAs in the prognosis and immune infiltration of breast cancer

**DOI:** 10.1038/s41598-022-25231-0

**Published:** 2022-11-30

**Authors:** Guo Huang, Hong Cao, Guowen Liu, Juan Chen

**Affiliations:** 1 Hengyang Medical School, University of South China, Hengyang, 421001 Hunan People’s Republic of China; 2 Key Laboratory of Tumor Cellular and Molecular Pathology, College of Hunan Province, Cancer Research Institute, University of South China, Hengyang, 421001 Hunan People’s Republic of China; 3 Department of Breast and Thyroid Surgery, Hengyang Medical School, The Second Affiliated Hospital, University of South China, Hengyang, 421001 Hunan China; 4 Department of Thyroid and Breast Surgery, The First Affiliated Hospital of Shenzhen University, Shenzhen Second People’s Hospital, Shenzhen, 518035 Guangdong China; 5 Department of Radiotherapy, The Second Affiliated Hospital, Hengyang Medical School, University of South China, Hengyang, 421001 Hunan China

**Keywords:** Oncology, Risk factors

## Abstract

Androgen receptor (AR) is strong association with breast cancer (BRCA). We aimed to investigate the effect of the androgen receptor signaling pathway-related long non-coding RNAs (ARSP-related lncRNAs) on the process of subtype classification and the tumor microenvironment (TME) of breast cancer (BRCA). Our study screen ARSP-related lncRNAs for the construction of a risk model. The single-sample gene set enrichment analysis (ssGSEA) method was used to detect the differences between the immune responses generated by the patients belonging to the low- and high-risk groups. The relationship between the ARSP-related lncRNAs and TME was explored following the process of cluster analysis. The univariate Cox analysis and the Lasso regression analysis method was used to screen nine of these lncRNAs to develop a risk model. It was observed that risk score could function as an independent prognostic factor, affecting the prognoses of patients suffering from BRCA. The validity of the model was assessed by analyzing the generated calibration curves and a nomogram. Additionally, the effect of the risk score on the extent of immune cell infiltration realized in TME was explored. M2 macrophages correlated positively, whereas NK cells, CD4+ T cells, and naive B cells correlated negatively with the risk score. Results obtained using the cluster analysis indicated that immune scores correlated with clustered subtypes. Finally, the risk score and cluster subtypes were analyzed to study the sensitivity of the patients toward different drugs to identify the appropriate therapeutic agents. The prognoses of patients suffering from BRCA can be accurately predicted by ARSP-related lncRNAs.

## Introduction

Breast cancer (BRCA) accounts for 25% of all new cancer cases, and it is the most prevalent cancer in women^1^. BRCA progression is closely related to estrogen and progesterone levels. Hormone receptor-positive (HR+) BRCA is the most prevalent type of cancer that accounts for over 70% of all BRCA cases^2^. Currently, 5 years of endocrine therapy is used to treat HR+ BRCA and reduce the extent of BRCA recurrence and associated death^3^. Additionally, the adjuvant endocrine therapy (duration: > 5 years) can help in the efficient reduction in the extent of BRCA recurrence and metastasis realized^4^. Although the use of systemic treatment regimens developed based on molecular typing and biological characteristics, have significantly improved the prognoses of patients with BRCA, recurrence or metastasis is still observed in some patients^5^. Therefore, the search for new treatment options has become the focus of current research.

Androgen receptor (AR) is expressed at varying levels in the ductal epithelium of normal breast ducts. A strong association is observed between AR and BRCA. Approximately 77% of the patients with BRCA exhibit AR expression and significant differences in the expression levels of different subtypes are observed^6^. AR can inhibit the process of estrogen-mediated cell proliferation and reduce BRCA incidence^7^. In Estrogen receptor positive (ER+) individuals, AR correlated positively with BRCA-specific survival, and under these conditions, the in vivo progression of ER+ BRCA was continuously inhibited^8^. However, high expression levels of AR, especially a high AR to ERα nuclear staining ratio (AR:ERα) in tumor cells, can result in resistance to endocrine therapy^9^. MCF-7 proliferation is dependent on AR signaling, and the AR antagonist enzalutamide (Enza) inhibit the growth of breast cancer xenograft tumors^10^. AR is associated with the overexpression of HER2. The HER2 signaling pathway promotes the activation of a downstream pathway associated with AR-related tumor growth to induce the proliferation of the HER2+ BRCA cells. However, the proliferative capacity is inhibited by the addition of androgen antagonists^11^. BRCA1 binds directly to the AR gene and affects the transcriptional activity of AR^12^. Furthermore, in the cases of ER+ BRCA that are characterized by the presence of BRCA1 mutations, the AR locus is extended by CAG repetitive sequences (> 27). It is associated with the attenuated expression levels of AR-regulated genes and an enhanced degree of cell proliferation^13^. As most patients with triple-negative breast cancer (TNBC) (60–80%) exhibit mutations in BRCA1 and AR activity is directly influenced by BRCA1, AR can potentially be a prognostic marker for TNBC^14^. Therefore, BRCA1 and AR can potentially exert a synergetic protective effect to realize the reduction of the incidence of BRCA.

Long non-coding RNAs (lncRNA) are non-protein-coding RNA molecules that are more than 200 nucleotides long. These can modify chromatin via the action of the lncRNA–protein or the generation of lncRNA–DNA interactions. Additionally, these undergo transcriptional activation or repression that are expressed via their interactions with transcriptional co-activators. These regulate protein stability and facilitate mRNA degradation. LncRNAs interact with miRNA and function as competing endogenous RNAs (ceRNA). These participate in the processes of mRNA phosphorylation, methylation, and ubiquitination. These are also associated with protein modifications^14^. The lncRNA–SLNCR1 binds to AR and blocks the SLNCR1–AR interaction in an androgen-independent manner, thus attenuating the invasion of SLNCR1-mediated melanoma^15^. The androgen-insensitive PCa cell lines were predominant in LINC00675. Under conditions of androgen deprivation, LINC00675 can bind to AR proteins to block the process of ubiquitination. In addition, GATA2 mRNA can also bind with LINC00675, resulting in the stabilization of the expression level. GATA2 can function as a coactivator of the AR signaling pathway in the nucleus. Additionally, the signaling axis corresponding to LINC00675/MDM2/GATA2/AR contributes to the generation of castration resistance. It also influences the process of progression of prostate cancer, acting as a therapeutic target^16^.

We aimed to determine the role of the androgen receptor signaling pathway (ARSP)-related lncRNAs in the incidence and progression of BRCA and their relationship with the tumor microenvironment (TME). Analysis of the prognostic model and the constructed subtypes help in the elucidation of the underlying mechanism following which the ARSP-related lncRNAs help generate the response of the patients toward immunotherapy.

## Material and methods

### Sources of data

**Figure 1:**
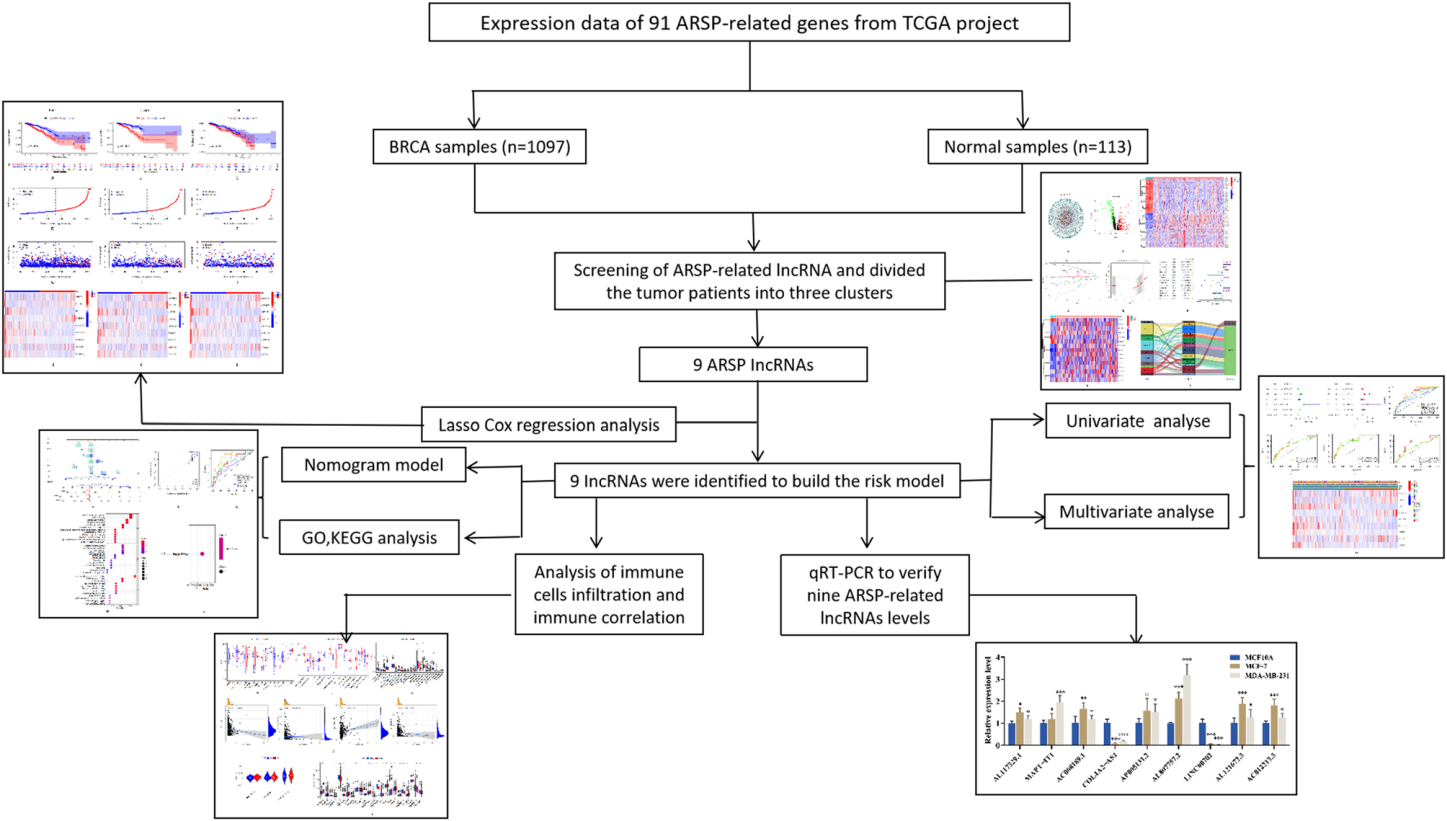
Study flow chart.

The Cancer Genome Atlas (TCGA) was analyzed to obtain the RNA sequencing (RNA-Seq) data for 1098 BRCA and 113 normal breast samples. The database can be accessed through https://portal.gdc.cancer.gov. The ComBat function was used to analyze all RNA-Seq data. The gene set enrichment analysis (GSEA) algorithm was used to obtain 91 ARSP-related genes. The online portal http://www.gsea-msigdb.org/gsea/ was accessed to obtain the required data (Table 1). The guidelines published for handling the TCGA database were followed to conduct the analyses. The work flow was shown in Fig. 1.

**Table 1:** Androgen receptor signaling pathway gene members.

Original member	Gene symbol	Gene description
100302237	MIR1281	MicroRNA 1281
1026	CDKN1A	Cyclin-dependent kinase inhibitor 1A
10273	STUB1	STIP1 homology and U-box containing protein 1
10399	RACK1	Guanine nucleotide binding protein (G protein), beta polypeptide 2-like 1
10401	PIAS3	Protein inhibitor of activated STAT, 3
10498	CARM1	Coactivator associated arginine member 1
10499	NCOA2	Nuclear receptor coactivator 2
10524	KAT5	Lysine acetyltransferase 5
106821730	BUB1B-PAK6	BUB1B-PAK6 readthrough
11034	DSTN	Destrin (actin depolymerizing factor)
11143	KAT7	Lysine acetyltransferase 7
11315	PARK7	Parkinson protein 7
1385	CREB1	cAMP responsive element binding protein 1
1387	CREBBP	CREB binding protein
1499	CTNNB1	Catenin beta 1
1616	DAXX	Death domain associated protein
166	TLE5	TLE family member 5
1956	EGFR	Epidermal growth factor receptor
2033	EP300	E1A binding protein p300
207	AKT1	AKT serine/threonine kinase 1
2119	ETV5	ETS variant transcription factor 5
2274	FHL2	Four and a half LIM domains 2
2288	FKBP4	FKBP prolyl isomerase 4
23028	KDM1A	Lysine demethylase 1A
2308	FOXO1	Forkhead box O1
2316	FLNA	Filamin A
23411	SIRT1	Sirtuin 1
23598	PATZ1	POZ/BTB and AT hook containing zinc finger 1
24149	ZNF318	Zinc finger protein 318
25942	SIN3A	SIN3 transcription regulator family
2932	GSK3B	Glycogen synthase kinase 3 beta
29893	PSMC3IP	PSMC3 interacting protein
3065	HDAC1	Histone deacetylase 1
354	KLK3	Kallikrein related peptidase 3
367	AR	Androgen receptor
3725	JUN	Jun proto-oncogene
387	RHOA	Ras homolog family member A
388	RHOB	Ras homolog family member B
3985	LIMK2	LIM domain kinase 2
4088	SMAD3	SMAD family member 3
4089	SMAD4	SMAD family member 4
4193	MDM2	MDM2 proto-oncogene
5052	PRDX1	Peroxiredoxin 1
51588	PIAS4	Protein inhibitor of activated STAT, 4
5295	PIK3R1	Phosphoinositide-3-kinase, regulatory subunit 1
5296	PIK3R2	Phosphoinositide-3-kinase, regulatory subunit 2
56924	PAK6	p21 (RAC1) activated kinase 6
57178	ZMIZ1	Zinc finger MIZ-type containing 1
5728	PTEN	Phosphatase and tensin homolog
573	BAG1	BAG cochaperone 1
5747	PTK2	Protein tyrosine kinase 2
5879	RAC1	Rac family small GTPase 1
5883	RAD9A	RAD9 checkpoint clamp component A
5901	RAN	"RAN, member RAS oncogene family
5925	RB1	RB transcriptional corepressor 1
595	CCND1	Cyclin D1
5970	RELA	RELA proto-oncogene
6013	RLN1	relaxin 1
6047	RNF4	Ring finger protein 4
6049	RNF6	Ring finger protein 6
6093	ROCK1	Rho-associated, coiled-coil containing protein kinase 1
64800	EFCAB6	EF-hand calcium binding domain 6
6605	SMARCE1	SWI/SNF related, matrix associated, actin dependent regulator of chromatin, Subfamily e, member 1
6667	SP1	Sp1 transcription factor
6714	SRC	SRC proto-oncogene
672	BRCA1	BRCA1 DNA repair associated
6774	STAT3	Signal transducer and activator of transcription 3
7041	TGFB1I1	Transforming growth factor beta 1
7050	TGIF1	TGFB induced factor homeobox 1
7182	NR2C2	Nuclear receptor subfamily 2, group C, member 2
7329	UBE2I	Ubiquitin-conjugating enzyme E2I
7337	UBE3A	Ubiquitin protein ligase E3A
7341	SUMO1	Small ubiquitin like modifier 1
8031	NCOA4	Nuclear receptor coactivator 4
811	CALR	Calreticulin
8202	NCOA3	Nuclear receptor coactivator 3
8431	NR0B2	Nuclear receptor subfamily 0, group B, member 2
8554	PIAS1	Protein inhibitor of activated STAT, 1
857	CAV1	caveolin 1
860	RUNX2	RUNX family transcription factor 2
8611	PLPP1	Phospholipid phosphatase 1
8648	NCOA1	Nuclear receptor coactivator 1
8850	KAT2B	Lysine acetyltransferase 2B
898	CCNE1	Cyclin E1
90427	BMF	Bcl2 modifying factor
9063	PIAS2	Protein inhibitor of activated STAT, 2
9475	ROCK2	Rho-associated, coiled-coil containing protein kinase 2
9604	RNF14	Ring finger protein 14
9611	NCOR1	Nuclear receptor corepressor 1
9612	NCOR2	Nuclear receptor corepressor 2
998	CDC42	Cell division cycle 42

### Model construction and the identification of the ARSP-related lncRNAs

The univariate Cox analysis method was used to screen the 91 ARSP-related lncRNAs and determine their influence on the prognoses of patients suffering from BRCA. All patients were randomly divided into two groups (training (n = 520) and test (n = 519)) in a 1:1 ratio. A tenfold cross-validation test was performed using the Lasso Cox regression algorithm using "glmnet" (R package) to construct a risk prediction model^17^. The multivariate Cox analysis approach was used to select the candidate genes and calculate the ARSP-related lncRNAs riskscore. The riskscore was determined as follows: riskscore = Σ (Expi × coefi), where Expi and Coefi represent the expression of each gene and risk coefficient, respectively. The median riskscore was analyzed to divide the patients into low-risk and high-risk groups. Finally, the receiver operating characteristic (ROC) curves were plotted following the Kaplan–Meier survival analysis method.

### Risk score model: construction and validation

The "survival" and "survival ROC" packages (R packages) were used to determine the predictive power of the model. The prognostic impact of the clinicopathological characteristics and risk scores were analyzed following the Kaplan–Meier survival and univariate and multivariate Cox regression analyses methods. The ROC curve was also analyzed to arrive at the results. Subsequently, the correlation between the expression levels of the nine ARSP-related lncRNAs and the clinicopathological characteristics was analyzed. A nomogram^18^ was constructed using the 'rms' package and assessed by analyzing the ROC curves corresponding to the 1-, 3-, and 5-year survival values. The predicted and actual 1-, 3-, and 5-year survival values were compared by analyzing the calibration plots.

### Functional enrichment analysis

Kyoto Encyclopedia of Genes and Genomes^19^ (KEGG) functional enrichment analyses and Gene Ontology (GO) enrichment analyses of the target lncRNAs were conducted to identify the potential functions and pathways associated with the high- and low-risk groups.

### Relationship between the risk models and TME and immune checkpoints

The single-sample GESA^20^ (ssGSEA) analysis method was used to determine the enrichment scores of different immune cell clusters belonging to the low- and high-risk groups. The scores were determined by analyzing the results obtained using the functional enrichment analysis method to identify the activities and pathways associated with the processes and study the relationship between the immune status and risk scores. The immune infiltration status and immune function (for the data identified from TCGA) of the patients with BRCA were calculated using CIBERSORT^21^. Limma, scales, Wilcoxon signed-rank test, "ggplot2", and "ggtext" (R packages) were used to analyze the differences between the content of the immune-infiltrating cells in the two groups. Additionally, TME scores and immune checkpoint blockage were compared between the two groups using "ggpubr" (R package).

### Analysis of drug sensitivity and genetic mutation

The 'maftools' R package was used to generate the mutation annotation format^22^ (MAF) for the data obtained from the TCGA database. The process was used to identify the somatic mutations in the patients belonging to the low- and high-risk groups. In addition, the tumor mutation burden (TMB) scores for all the patients were also calculated. The half-inhibitory concentration (IC50) values for the chemotherapeutic agents commonly used to treat BRCA were determined using the pRRophetic^23^ package to compare the efficiencies of the chemotherapeutic agents used to treat the patients belonging to the two groups.

### Nine prognosis-related lncRNAs-based clusters

Potential molecular clusters were identified using the consensus clustering (CC) method and R package by analyzing the expression levels of the prognosis-related lncRNAs. The results were analyzed to understand the response of the patients toward drug therapy^15^. Various methods such as the principal component analysis (PCA), t-distribution stochastic neighbor embedding (t-SNE), and Kaplan–Meier analysis were used to predict patient survival rates. Immunoassays were conducted, and drug sensitivity was compared using the GSVA Base and “pRRophetic” R packages.

### Cell culture

MDA-MB-231 and MCF-7 cells were cultured in RPMI-1640 medium and MCF-10A cells in DMEM medium, all media were mixed with 10% fetal bovine serum + 1% double antibody. Cells were incubated in 5% CO_2_ in a 37 °C thermostat.

### Quantitative real-time PCR (qRT-PCR)

Total RNA was extracted from MCF-10A cell, MDA-MB-231 cell and MCF-7 cell using Total RNA Extraction Kit (Servicebio, China). To quantify nine ARSP-related lncRNAs levels, reverse transcription of cDNA was performed using prime script rt kit (Takara). The nine lncRNAs expression levels were measured using SYBR Green qPCR Mix (Takara). The primers sequences were listed Table 2. The relative expression levels of the eight pyroptosis-related lncRNAs were determined using the 2^−ΔΔCt^ method.

**Table 2:** Primer sequences for qRT-PCR.

Primer	Sequence (5′–3′)
AC012213.3-F	TAGAGGACATTGGAGGGGCA
AC012213.3-R	TTGGAGAAGTTGTGGCTGCA
AL117329.1-F	GTCAGAACAGGGAGGTCGTG
AL117329.1-R	CACGTGTCACTGTTGCCAAG
MAPT-IT1-F	GGCCACACCCATCTTTCTGA
MAPT-IT1-R	TTCAGATCAACCTGGGCGAC
AC068189.1-F	CAGTCGTGTGCTGAAATCCG
AC068189.1-R	GCCTGGACAACACAGTGAGA
COL4A2-AS1-F	GCCTAGAACCATCGCTCTCC
COL4A2-AS1-R	TAGGGATGGTGGAGGGGAAG
AP005131.2-F	AAGAGGGAGGCCATCTGGAT
AP005131.2-R	CCACAGCTCCTCTGATTGCA
AL807757.2-F	CTCCTACCTCAGCCTGGACT
AL807757.2-R	CATGCAATACGCTTGGCCTC
LINC00702-F	TTTCTCGTGTCTGAGGCACC
LINC00702-R	TGGCCCCATGGGTTTATTCC
AL121672.3-F	AGCCTGGGATGTCAAAGCTC
AL121672.3-R	TCACCAACGAGGAAGCTGAC

### Ethical approval

As this work is a bioinformatics analysis, ethical approval is not required. All methods were performed in accordance with the relevant guidelines and regulations.

### Statistical analysis

The ComBat function (sva package) was used to normalize all RNA-Seq data. The Wilcoxon rank-sum test was conducted to compare the gene expression levels observed in the tumor and normal tissues. The Kaplan–Meier method was used to plot the survival curves, and the method of cluster typing was executed using the consensus clustering package. The characteristics of the immune cells that infiltrated tumors were analyzed using the ssGSEA algorithm. The R language package was accessed through https://www.r-project, and the data were used for statistical analyses. P < 0.05 indicates a statistically significant difference.

## Results

### ARSP-related lncRNAs in patients with BRCA

**Figure 2:**
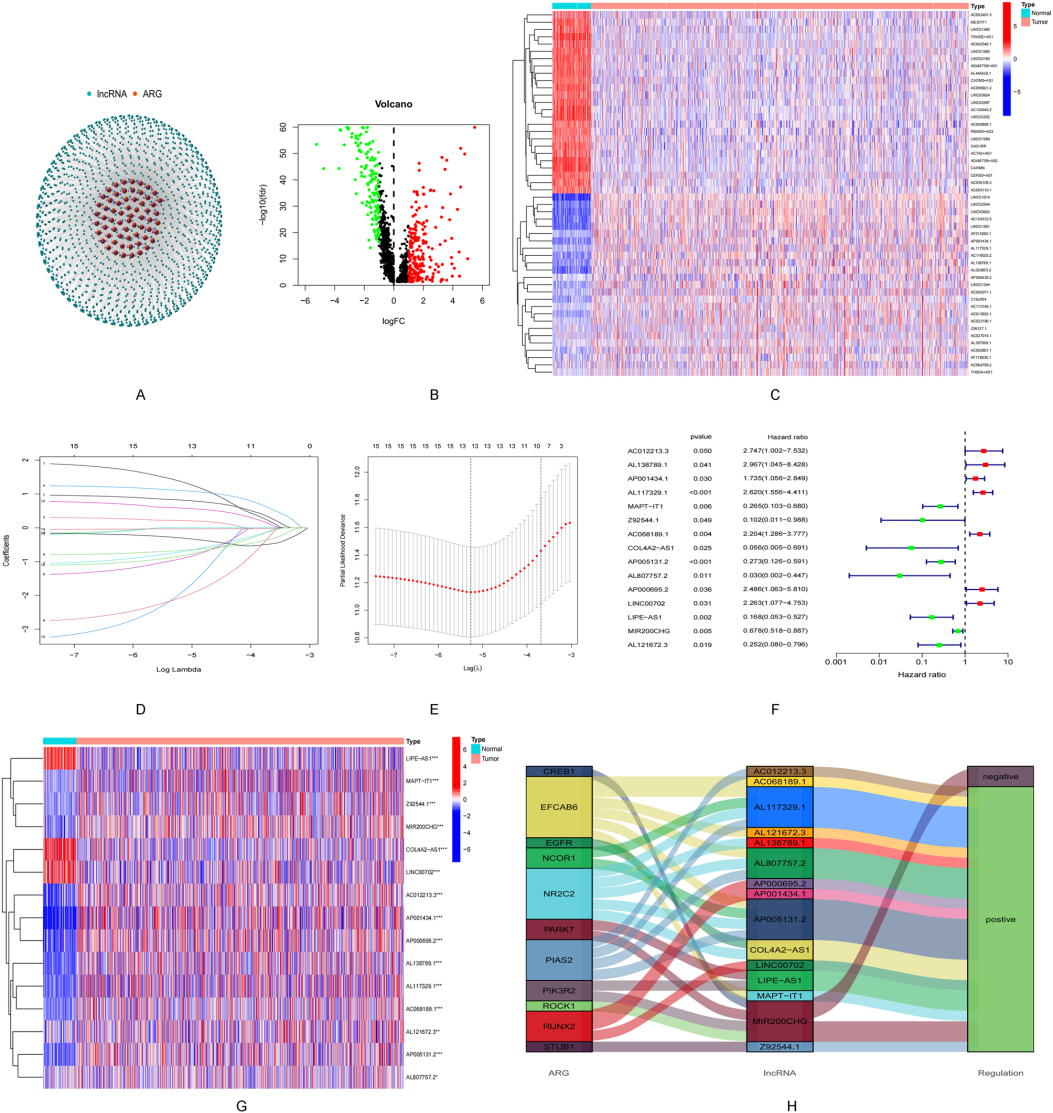
Identification of androgen receptor signaling pathway-related lncRNAs in breast cancer patients. (**A**) Network between necrotic genes and lncRNAs (correlation coefficients > 0.4 and P < 0.001). (**B**) Volcanic atlas of 340 differentially expressed ARSP genes. (**C**) The expression profiles of the 25 lncRNAs with the most significant high and low expressions. (**D**) 10 × cross-validation of variable selection in the LASSO model. (**E**) Lasso coefficient profiles of 15 ARSP-lncRNAs. (**F**) Prognostic lncRNA extracted by univariate Cox regression analysis. (**G**) Expression profiles of 15 prognostic lncRNAs. (**H**) Sankey diagram of ARPS genes and related lncRNAs.

A total of 340 ARSP-related lncRNAs were identified based on the expression levels of the differentially expressed lncRNAs and the 91 ARSP-related genes (|log2FC|> 1; P < 0.05) (Fig. 2A). Of these, up-regulation was observed for 203 lncRNAs, and down-regulation was observed for 137 lncRNAs (Fig. 2B). The heatmap presents the top 25 genes that were up-regulated and down-regulated (Fig. 2C). The Lasso regression analysis method was followed to screen 15 ARSP-related lncRNAs (Table 3) to construct a prognostic risk model (Fig. 2D,E), which was visualized using a forest plot (Fig. 2F) and a heat map (Fig. 2G). Analysis of the Sanger plot helps understand the ARSP-related genes and their relationship with lncRNAs (Fig. 2H).

**Table 3:** Univariate analysis showing associations between androgen receptor signaling pathway-related lncRNA in BRCA. Unadjusted HRs are shown with 95% confidence intervals.

Gene	HR	HR.95L	HR.95H	P value
AC012213.3	2.747322169	1.002147117	7.531607862	0.049514741
AL138789.1	2.967063297	1.044530094	8.428157946	0.041175869
AP001434.1	1.734643892	1.056221693	2.848823741	0.029550745
AL117329.1	2.619699853	1.555973505	4.410632506	0.000290924
MAPT-IT1	0.26463239	0.102913288	0.680478714	0.005800638
Z92544.1	0.101981797	0.010528208	0.987849705	0.048779306
AC068189.1	2.204187144	1.286184878	3.777404827	0.004031392
COL4A2-AS1	0.05649771	0.004616883	0.691373577	0.024525489
AP005131.2	0.272670097	0.125899035	0.590544494	0.000981316
AL807757.2	0.030066387	0.002022239	0.447023091	0.010940248
AP000695.2	2.485621742	1.063333134	5.810329097	0.035578713
LINC00702	2.262856038	1.077283191	4.753176779	0.031040643
LIPE-AS1	0.167655362	0.053302878	0.527332136	0.002254501
MIR200CHG	0.677878113	0.518308682	0.886573488	0.004523808
AL121672.3	0.252159371	0.079883616	0.795962316	0.018820507

### Model construction and validation

**Figure 3:**
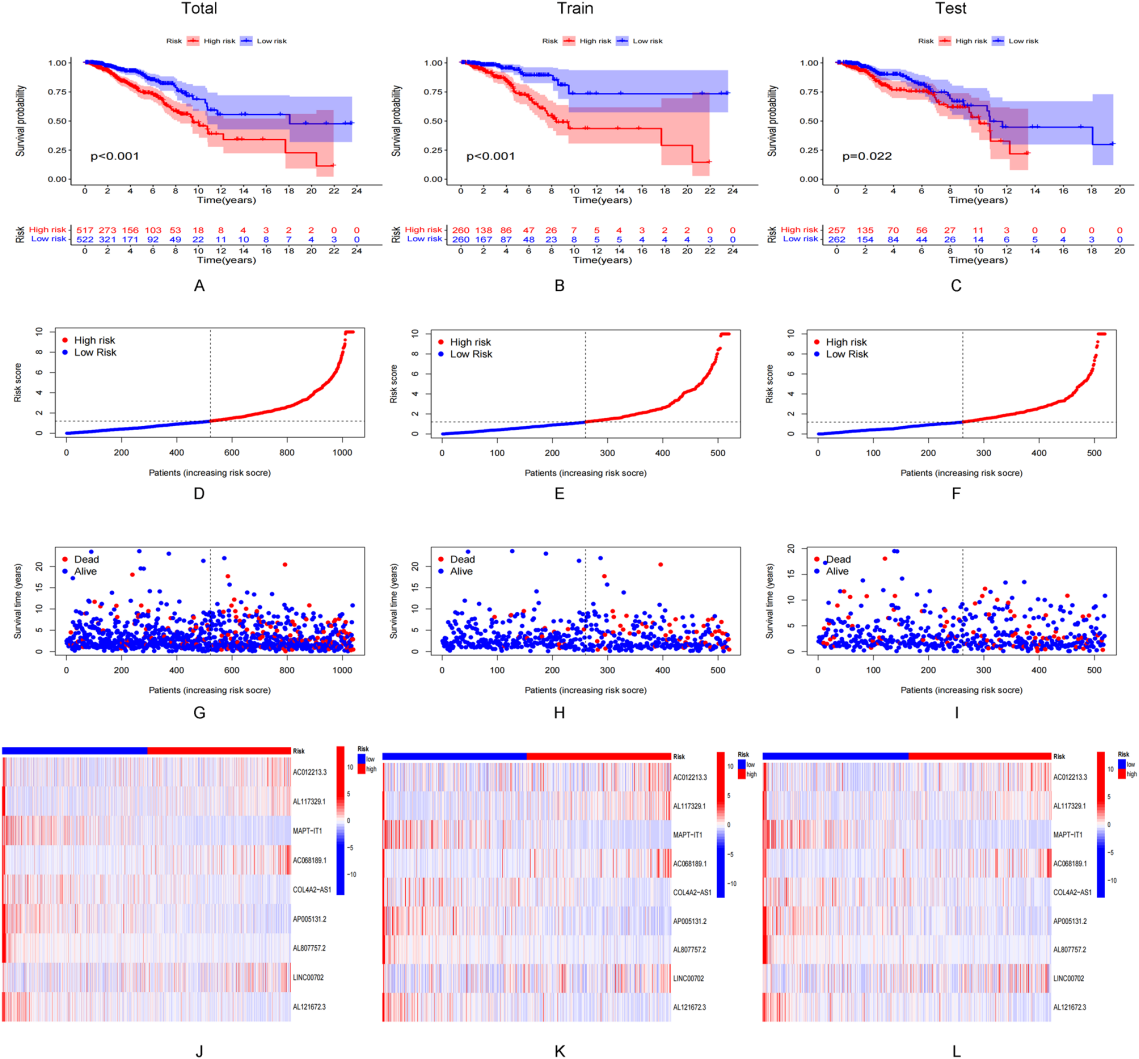
Prognostic value of 15 necrosis-associated lncRNAs models across the total dateset, training dateset, and validation dateset. (**A**–**C**) Kaplan–Meier survival curve based on OS (survival probability) of the total dateset, training dateset, and validation dateset. (**D**–**F**) Risk score for the otal dateset, training dateset, and validation dateset. (**G**–**I**) Survival time and survival status of the otal dateset, training dateset, and validation dateset of low- and high-risk groups. (**J**–**L**) Heat maps expressed by 9 lncrnas in the otal dateset, training dateset, and validation dateset.

**Figure 4:**
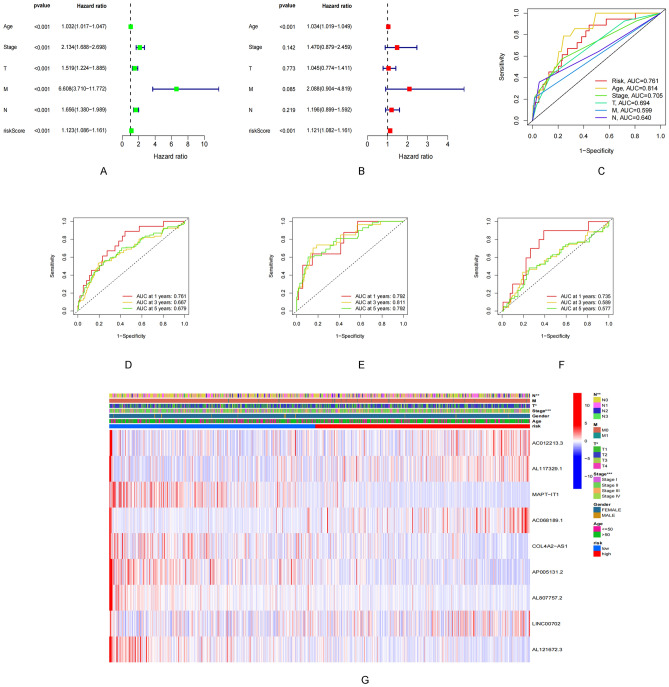
Evaluation and validation of the risk model. (**A**,**B**) Univariate and multifactor Cox analysis of risk score and clinical characteristics. (**C**) 1-year ROC curves for risk score and clinical characteristics. (**D**–**F**) 1-year, 3-year and 5-year ROC curves for the total dateset, training dateset, and validation dateset. (**G**) Differential expression of 9 ARSP-lncRNAs in different risk score and clinical features.

A prognostic risk model was constructed by screening 9 lncRNAs using the multivariate Cox regression analysis method. Results obtained following the Kaplan–Meier analysis method revealed that the survival potential of patients belonging to the low-risk group was significantly higher than the survival potential of the patients belonging to the high-risk group (Fig. 3A–C). The median risk score was 1.86 (Fig. 3D–F). It was also observed that the number of deaths in the high-risk group was higher than the number of deaths in the low-risk group (Fig. 3G–I). The expression levels of these nine lncRNAs belonging to both groups are presented in Fig. 3J–L. The results obtained using the multivariate and univariate Cox regression analyses methods revealed that the risk score was an independent prognostic factor (Fig. 4A–C). The ROC curves corresponding to the 1-, 3-, and 5-year OS were further analyzed to determine the predictive value of the risk model in the BRCA cohort (Fig. 4D–F). The results indicated a strong sensitivity and specificity for the model for survival prediction. The heat map demonstrates the relationship between the risk score and the nine lncRNAs and clinicopathological characteristics (Fig. 4G). MAPT–IT1, AP005131.2, AC012213.3, COL4A2–AS1, and AL807757.2 in the risk model correlated strongly with the prognoses of patients with BRCA (Supplementary Fig. 1). It was observed that the prognosis realized for the patients in the low-risk group was significantly better than the prognosis realized for the patients in the high-risk group. This was validated by the differences in age, gender, T1–2, N0/N1-3, M0, and Stage I–II/Stage III–IV (Supplementary Fig. 2).

Risk score = (expression of AC012213.3 × 1.949) + (expression of AL117329.1 × 1.318) + (expression of MAPT-IT1 × − 1.189) × (expression of AC068189.1 × 0.813) + (expression of COL4A2-AS1 × − 2.417) + (expression of AP005131.2 × − 0.803) + (expression of AL807757.2 × − 3.857) + (expression of LINC00702 × 0.708) + (expression of AL121672.3 × − 1.502).

### Construction of the pyroptosis risk score-based nomogram

**Figure 5:**
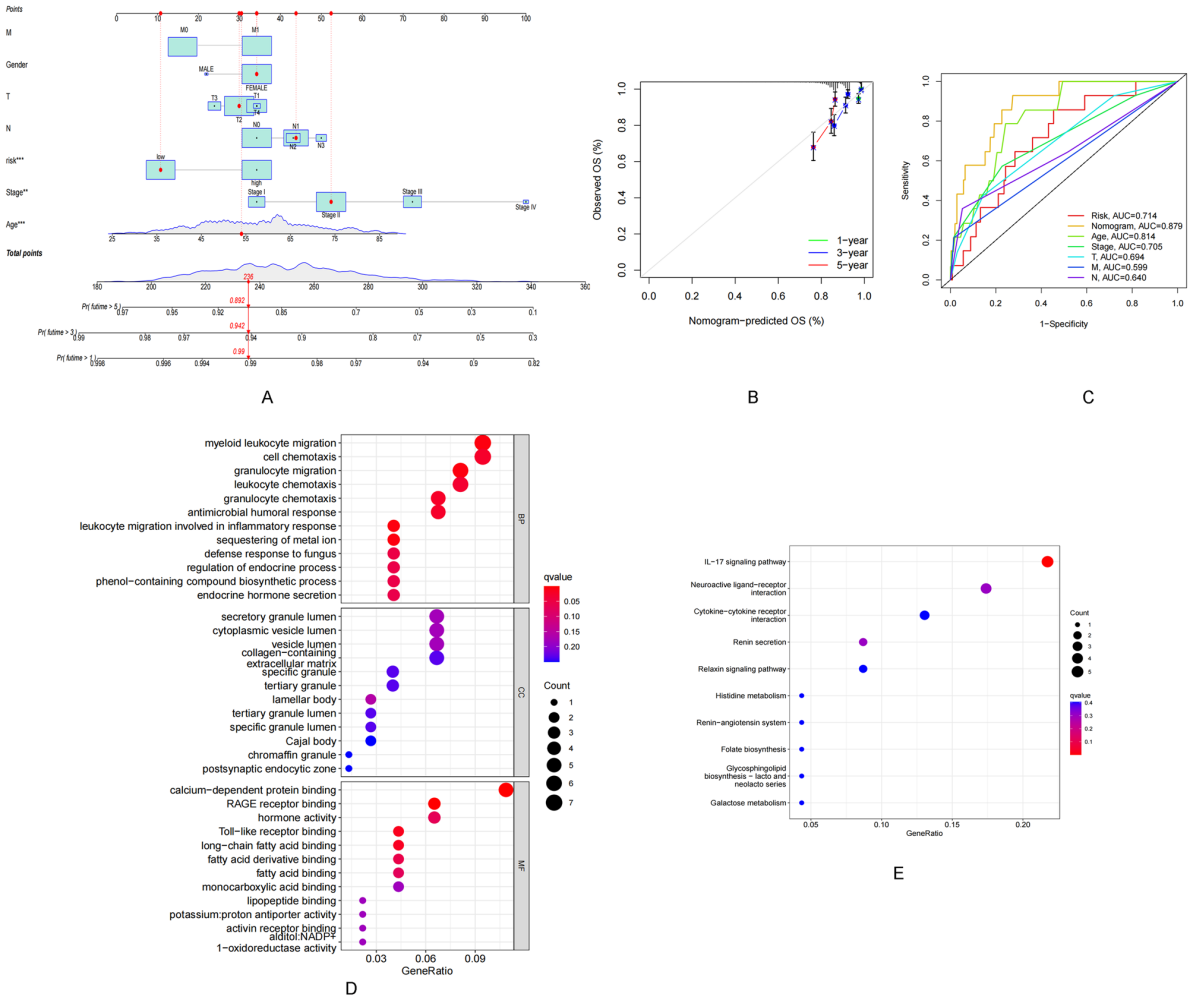
Nomogram and assessment of the risk model. (**A**) Nomogram predicting the probability of 1-year, 3-year, and 5-year OS for risk score and clinical characteristics. (**B**) Calibration curves of the nomogram for predicting of 1-, 3-, and 5-year OS in all BRCA patients. (**C**) ROC curves for risk score, Nomogram score, and clinical characteristics. (**D**,**E**) GO and KEGG enrichment analysis of risk score-associated genes.

A prognostic nomogram based on risk score and clinicopathological characteristics was constructed to predict the prognoses of patients with BRCA (Fig. 5A). The calibration curve was approximately diagonal. This indicated that the predictive power of the nomogram for 1-, 2-, and 3-year OS was strong (Fig. 5B). Therefore, it can be inferred that both risk score and nomogram exhibit good predictive power (Fig. 5C).

### Functional enrichment analysis of risk score

Risk score-related genes were subjected to GO and KEGG enrichment analyses. The results revealed that the primary biological processes occurring included myeloid leukocyte migration, granulocyte migration, leukocyte migration associated with inflammatory response, sequestering of metal ion, and granulocyte chemotaxis (Fig. 5D). In addition, the results obtained using the KEGG pathway analysis method suggested a significant enrichment in the IL-17 pathway. Neuroactive ligand-receptor interaction and Cytokine–cytokine receptor interaction (Fig. 5E).

### Analysis of immune status and TME

**Figure 6:**
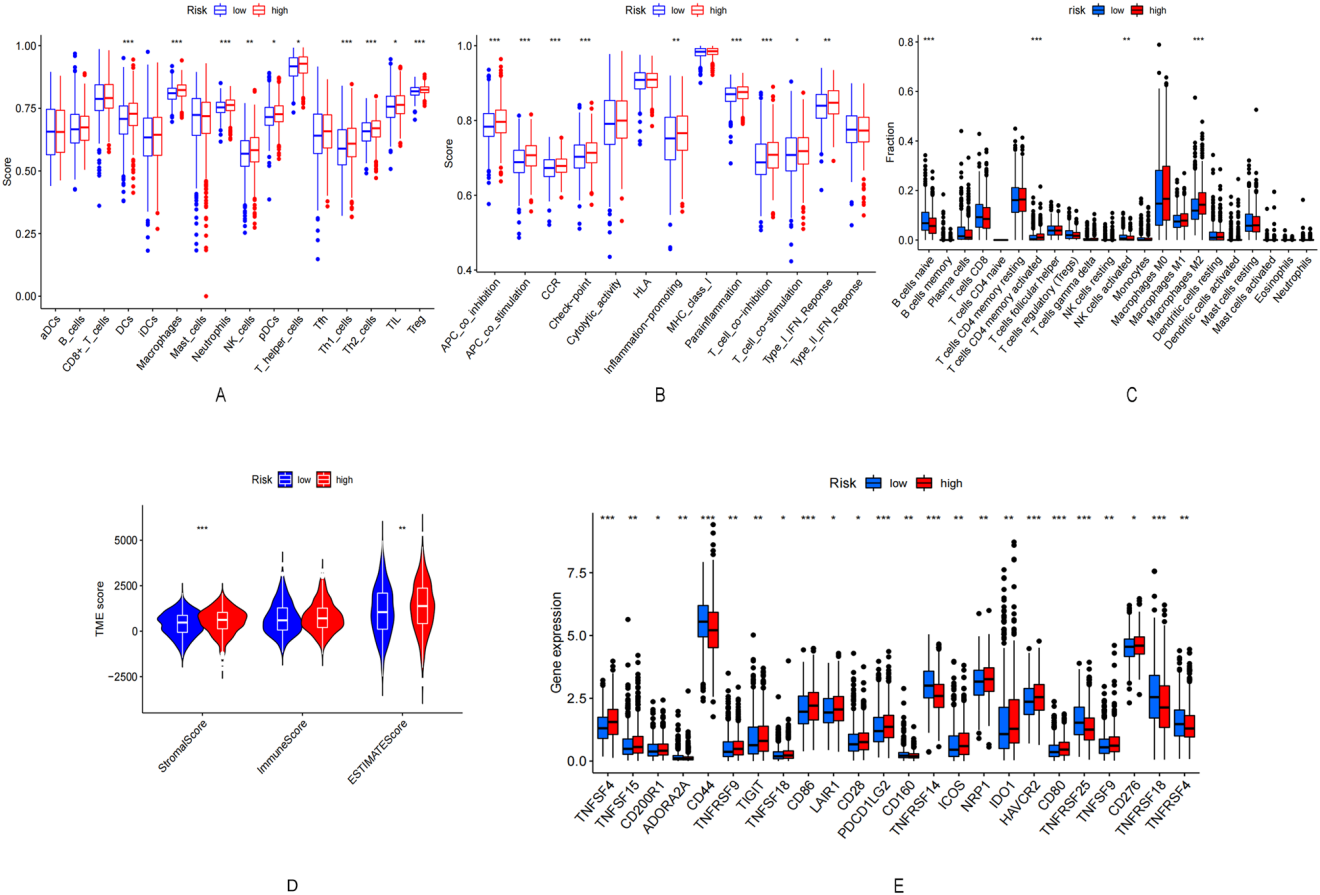
Risk score associated with immune cell infiltration and function. (**A**,**B**) ssGSEA scores of immune cells and immune function in the high/low risk group. (**C**) Distribution of immune cell infiltration in the high/low risk group. (**D**) Correlation between high/low risk group and immune cells. (**E**) Comparison of high/low risk groups in immune correlation scores. (**F**) Differences in immune blocking site expression in high/low risk groups.

The enrichment scores corresponding to different immune cell clusters, related functions, and pathways were quantified using ssGSEA to study the relationship between the risk score and immune status. The levels of DC, NK cells, pDCs, TIL, Th2 cells, Treg cells, Th1 cells, APC co-stimulation, APC co-inhibition, T cell co-inhibition, and T cell co-stimulation in the high-risk group (Fig. 6A,B) were significantly higher than their corresponding levels in the low-risk group. Additionally, levels of CCR, checkpoints, macrophages, neutrophils, inflammation-promoting activity, and type I IFN reactivity scores in the high-risk group were higher than the corresponding levels in the low-risk group (adjusted P < 0.05).

The relationship between risk score and immune infiltration was determined to explore the relationship between risk score and immune components. Naive B cells, CD4T memory cells, M2 macrophages, and activated immune-infiltrating NK cells could be correlated with the risk score (Fig. 6C). Further analysis suggested that the risk score could be used as an indicator of immunity. Low TME scores correlated strongly with high immune scores, whereas high TME scores correlated strongly with high stromal scores (Fig. 6D). Most immune checkpoints were highly activated in patients belonging to the high-risk group (Fig. 6E). These findings guide us in the selection of appropriate checkpoint inhibitors for different patients based on risk groups. The IC50 of the chemical or targeted agents (used to treat BRCA), such as Nilotinib, Gefitinib, Epothilone. B, Elesclomol, Bosutinib, and Lenalidomide were lower in the low-risk group than the IC50 levels of these agents in the high-risk group. It was also observed that the IC50 of the androgen receptor inhibitor Bicalutamide was lower in the high-risk group than the IC50 of the same inhibitor in the low-risk group (Supplementary Fig. 3).

### Relationship between risk score and TMB

**Figure 7:**
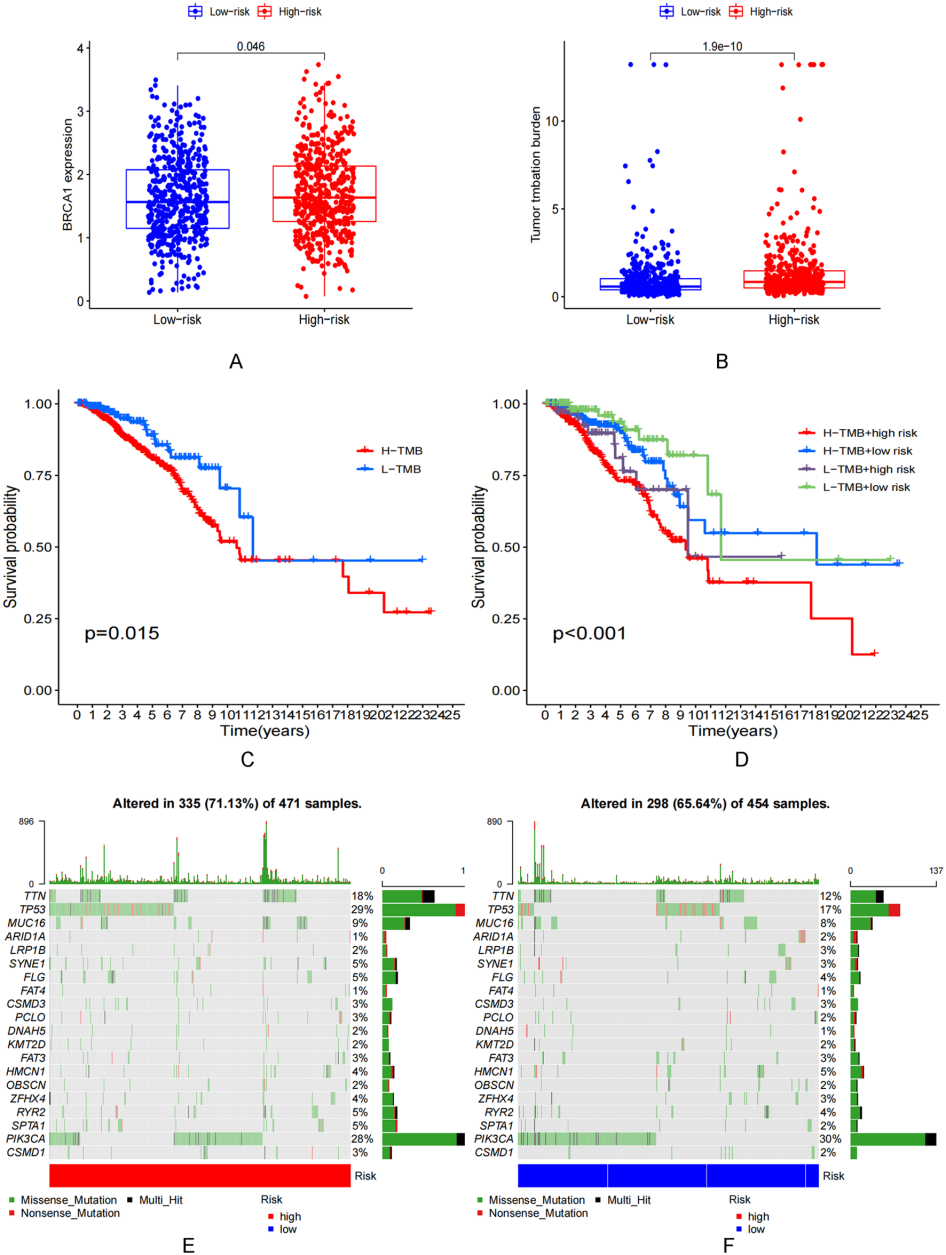
Construction of the ARSP-lncRNAs risk score. (**A**) BRCA1 expression in high and low risk group. (**B**) Correlation of BRCA1 with risk score. (**C**) TMB status in high and low risk group. (**D**) Correlation of TMB with risk score. (**E**) Prognostic analysis of TMB. (**F**) Prognostic analysis of TMB with high and low risk group. (**G**,**H**) Waterfall plots of somatic mutation characteristics in high and low mortality scores. Each column represents an individual patient.

The expression of breast cancer susceptibility gene 1 (BRCA1) could be related to the extent of BRCA progression realized. The level of expression was significantly high in the high-risk group (Fig. 7A). TMB was significantly high in the high-risk group (Fig. 7B). It was observed that the OS recorded for the patients with high TMB was poorer than the OS recorded for patients with low TMB (Fig. 7C). The best prognoses were observed for patients characterized by low-risk score and low TMB (Fig. 7D). PIK3CA and TP53 were characterized by the maximum mutation frequencies in the low- and high-risk groups, respectively (Fig. 7E,F).

### TME subtypes in the risk model

**Figure 8:**
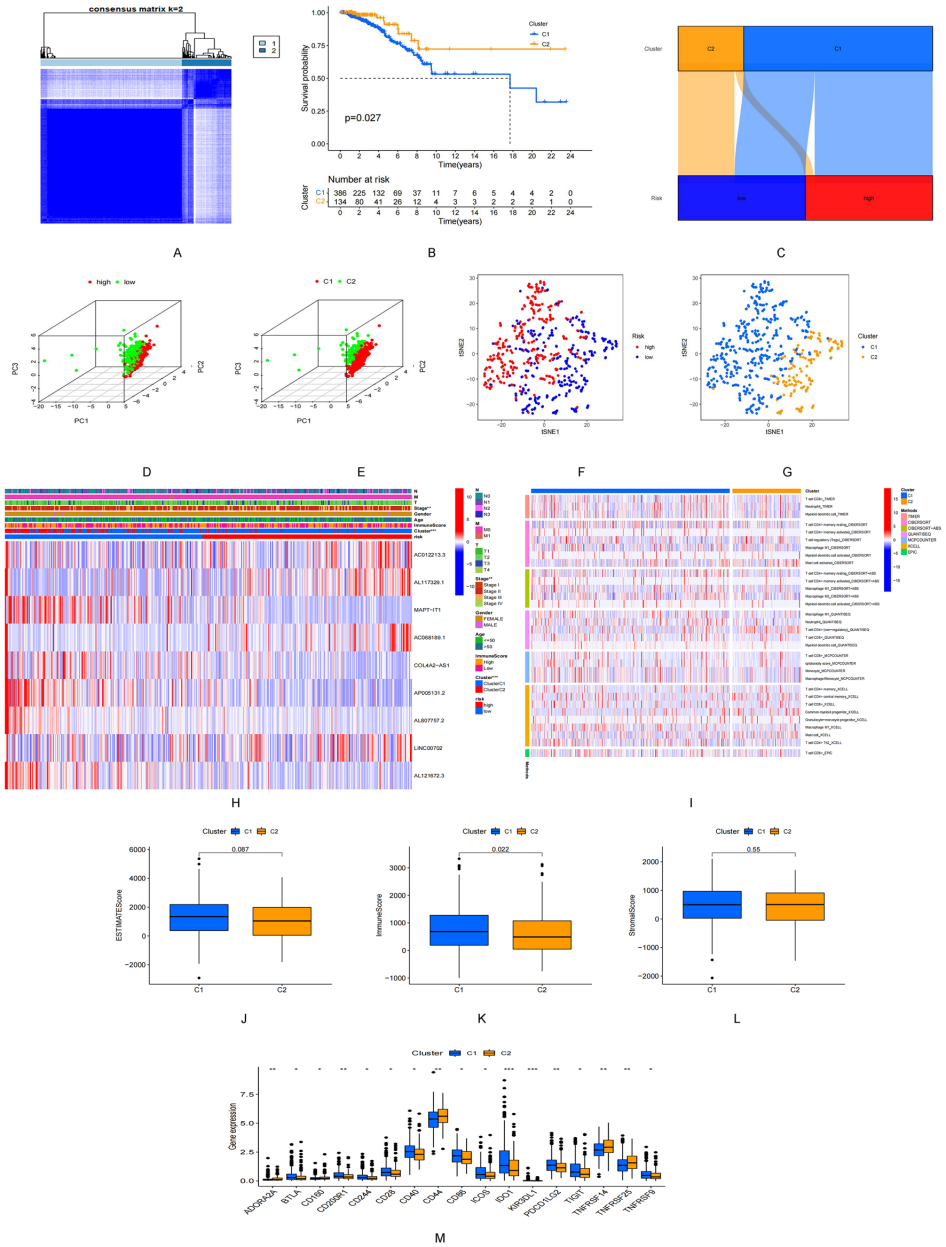
ARSP-lncRNAs subtypes and clinical characteristics. (**A**) Consensus matrix heat map defining the three clusters (k = 2). (**B**) Kaplan–Meier survival curves for the three subtypes. (**C**) Sankey diagram of three subtypes and high and low risk group. (**D**,**E**) PCA differentiation between high- and low-risk groups and three subtypes. (**F**,**G**) tSNE differentiation between high- and low-risk groups and three subtypes. (**H**) Differences in expression of ARSP-lncRNAs between the three subtypes, high- and low-risk and clinicopathological features. (**I**) Heat map of immune cell infiltration in the three subtypes. (**G**–**L**) Correlation of different subtypes with ESTIMATE score, Immune score and Stromal score. (**M**) Differences in expression of immune blocking sites in different subtypes.

Patients were reclassified into three clusters using the CC method and R package based on the nine ARSP-related lncRNAs (Fig. 8A). The results from the Kaplan–Meier analysis revealed that excellent OS could be achieved for patients belonging to Cluster C2 (P = 0.027, Fig. 8B). Most the patients in Cluster C2 belonged to the low-risk group, whereas the patients in Cluster C1 mostly belonged to the high-risk group (Fig. 8C). Results from PCA suggested that both the risk groups and the three clusters were characterized by different PCs (Fig. 8D,E). The tSNE method could be used to accurately distinguish the patients in both the risk groups by analyzing the three clusters (Fig. 8F,G). Analysis of the heatmap revealed the differential expression of the nine lncRNAs (with respect to tumor size, lymph node metastasis, tumor stage, and clustered subtypes) in the samples belonging to the low and high-risk groups (Fig. 8H). Different immune cell infiltration platforms were analyzed, and the results suggested that the patients in Cluster C1 were characterized by a high degree of immune cell infiltration (Fig. 8I), the Cluster C1 have maximum ESTIMATE scores, Immune score and Stromal score (Fig. 8J–L). All immune checkpoints, including PDCD1LG2, TIGIT, and IDO1, were differentially expressed in different clusters (Fig. 8M).

### Sensitivity of patients belonging to different clusters toward immunotherapy

The clustering analysis method was used to reveal that the patients in Cluster C1 and C2 exhibited the best sensitivity toward drug therapy. It was also observed that the IC50 of Docetaxel, Rapamycin, Sorafenib, Vinblastine and Salubrinal were the maximum in Cluster C2, while the IC50 of Elesclomol and Nilotinib were the maximum in Cluster C1 (Supplementary Fig. 4A–G).

### Validated these eight genes by cellular experiments

**Figure 9:**
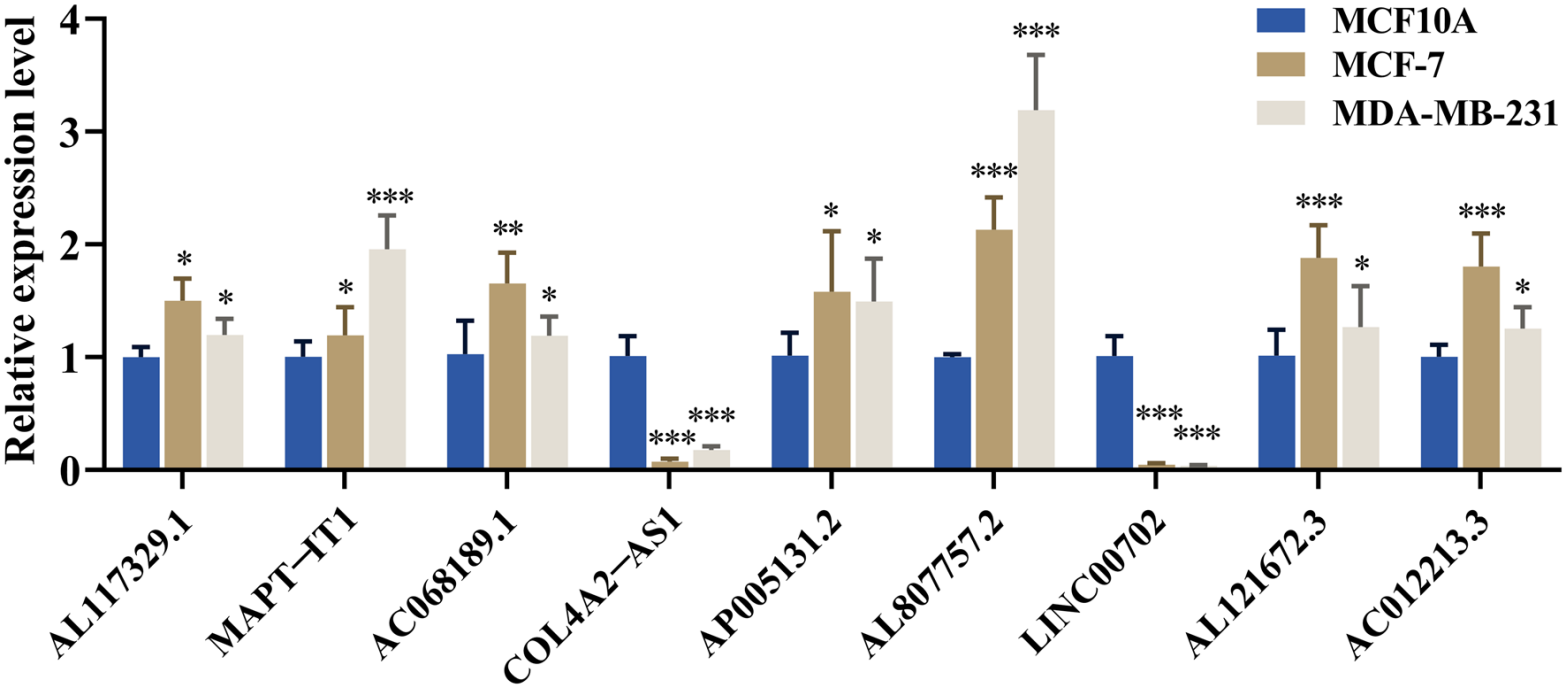
The relative expression levels of the nine lncRNAs in MCF-10A, MCF-7 and MDA-MB-231 by qRT-PCR (*P < 0.05; **P < 0.01, ***P < 0.001).

The qRT-PCR was performed on mammary epithelial cell MCF-10A, breast cancer cells MDA-MB-231 and MCF-7 to verify the mRNA expression levels of these nine characteristic genes. All results were in general agreement with the data in TCGA (Fig. 9) (*P < 0.05; **P < 0.01, ***P < 0.001).

## Discussion

We constructed a risk model by analyzing nine ARSP-related lncRNAs to predict the prognoses of patients suffering from BRCA. The model exhibited a good predictive power. The risk score was an independent prognostic factor characterized by good specificity and sensitivity. Both the risk model and clustered subtypes could be analyzed to effectively distinguish between the clinical characteristics of patients belonging to the low- and high-risk groups. A strong association between the high-risk score and the regulation of immune function and the extent of immune cell infiltration realized was also observed. Therefore, these findings suggest that the risk model influences the TME in patients with BRCA.

BRCA is the most prevalent malignancy in women worldwide and poses a great threat to women's health. More than 1.2 million people die due to BRCA each year in China, accounting for 9. 6% of global BRCA deaths^24^. BRCA is a hormone-related tumor. The AR signaling pathway significantly affects the progression of BRCA. AR, a transcription factor activated by androgens (e.g., testosterone in men and dihydrotestosterone in women), is differentially expressed in different BRCA subtypes, and it can potentially affect the OS of patients with BRCA^25^.

LncRNA AC012213.3 is highly expressed in BRCA tissues and cell lines and can be correlated with poor prognosis and clinical characteristics. The overexpression of AC012213.3 can potentially result in enhanced proliferation of BRCA cells and BRCA cell invasion. These are realized through the promotion of the process of transcription of the downstream target gene RAD54B^26^. MAPT–IT1 is associated with Koolen-De Vries syndrome ^27^ and can potentially predict the prognoses of patients with BRCA^28^. It was observed that the expression levels of COL4A2–AS1 in colorectal cancer tissues and cells were up-regulated. This promoted the processes of aerobic glycolysis and cell proliferation, affecting the process of in vivo tumor growth. The primary mechanism can potentially involve the sponge-adsorption of COL4A2–AS by miR-20b-5p. This helps regulate the HIF1A expression levels in colorectal cancer^29^. It has been reported that low COL4A2–AS1 expression levels can be potentially associated with better OS in patients with BRCA. However, its role as a diagnostic and prognostic indicator in BRCA should be further studied^30^. AP005131.2 can potentially function as a novel biomarker and therapeutic target for BRCA in a risk model constructed based on m5C-lncRNAs that are associated with the processes of tumor immune cell infiltration and cancer metabolism^31^. LINC00702 can potentially inhibit the processes of cell growth and metastasis by regulating the expression phosphatase and tensin homolog deleted on chromosome ten (PTEN) levels in colorectal cancer^32^. LINC00702 can potentially function as a biomarker for the prediction of the prognoses of patients suffering from malignant meningioma. It can also function as an oncogene during the progression of malignant meningioma by regulating the miR-4652-3p/ZEB1 axis and activating the Wnt/β-catenin signaling pathway^33^. However, the roles of AL117329.1, AC068189.1, AL807757.2, and AL121672.3 in the incidence and progression of BRCA are not reported.

We also note that in breast cancer research, the high expression of HOTAIR is closely related to breast cancer lymph node metastasis (LNM), and there is a direct strong correlation with the expression of androgen receptor (AR) These data confirm that HOTAIR is involved in the regulation of AR pathway, which provides the possibility for AR-positive TNBC patients to establish new treatment strategies^34^. Recent studies have found that AR negatively regulates lncRNA-ARNILA, which is associated with poorer progression-free survival (PFS) in TNBC patients, promotes epithelial cell-mesenchymal transformation (EMT), invasion, and metastasis. lncRNA-ARNILA, as a competitive endogenous RNA (ceRNA) for miR-204, promotes the expression of its target gene Sox4, induces EMT and promotes breast cancer progression^35^.

Results from KEGG functional enrichment analysis revealed a close association between the IL-17 signaling pathway and the progression of BRCA. It was also observed that HIF1α could influence the sensitivity of patients toward paclitaxel chemotherapy by regulating the IL17 signaling pathway^36^. Enhanced PD-1/PD-L1 expression was associated with the upregulation of the IL-17 signaling pathway-related genes, and the improved IL-17 signaling was found to be associated with the elevated extents of CD8+ T cell infiltration and changes in TME in patients with BRCA^37^.

The results reported herein reveal that the risk model constructed by analyzing the ARSP-related lncRNAs was strongly associated with different immune cell clusters and immune cell infiltration levels. M2 macrophages correlated positively with the risk score. Low TME scores were strongly associated with high immune scores, whereas high TME scores were strongly associated with high stromal scores. Thus, there may be a close relationship between the ARSP-related lncRNA and TME^38^. Androgens are produced locally in BRCA tissues by androgen-secreting enzymes (such as 5α-reductase type 1), which act on both BRCA cells and TME. Tumor-associated macrophages (TAMs) are a major component of the TME and contribute to tumor progression^39^. Although the androgen/AR signaling pathway in macrophages plays a crucial role in the progression of human disease, the role of androgens regulatory effect of TAMs remains largely unknown. The expression of AR within macrophages in tumors can potentially promote the growth of tumors and help increase the ki67 expression level, resulting in enhanced tumor invasive properties, suggesting that androgens can potentially improve the ability of macrophages to promote BRCA progression^40^.

## Conclusion

We are the first to identify the ARSP-related lncRNAs and develop a model for predicting the prognoses of patients suffering from BRCA. We observed that risk score and clustered subtypes were associated with TME and the expression levels of immune checkpoint molecules. Overall, ARSP can serve as a therapeutic target to improve the immunotherapeutic effect associated with BRCA. The applications of the results are restricted by the limitations of the study. The data required to conduct the experiments were obtained from the TCGA database. Therefore, in vivo or in vitro baseline trials should be carried out in the future to confirm the applicability of the AR inhibitors and immune checkpoint inhibitors in the field of developing ARSP-related drug combinations and BRCA treatment methods. Based on these results, we will further investigate the response of the patients toward targeted therapy and immunotherapy techniques in the future to identify and develop precise treatment methods for BRCA.

## Supplementary Information

Supplementary Figures.

## Data Availability

The datasets used and/or analyzed during the current study available from the corresponding author on reasonable request.

## Abbreviations

BRCA: Breast cancer
ARSP: Androgen receptor signaling pathway
ER+: Estrogen receptor positive
TMB: Tumor mutation burden
UCSC: The University of California, Santa Cruz
TCGA: The Cancer Genome Atlas
GEO: Gene Expression Omnibus
TME: Tumor microenvironment
GSEA: Gene set enrichment analysis
TNBC: Triple negative breast cancer
DEGs: Differentially expressed genes
PCA: Principal component analysis
AUC: Area under the curve
ROC: Receiver operating characteristic curve
ssGSEA: Single sample gene set enrichment analysis
GO: Gene ontology
KEGG: Kyoto encyclopedia of genes and genomes
OS: Overall survival
IC50: Half maximal inhibitory concentration
TAMs: Tumor-associated macrophages

## References

[1] Erratum: Global cancer statistics 2018: GLOBOCAN estimates of incidence and mortality worldwide for 36 cancers in 185 countries. *CA Cancer J. Clin.* **70**, 313. 10.3322/caac.21609 (2020). 10.3322/caac.21609 32767693

[2] Gianni L (2014). Neoadjuvant and adjuvant trastuzumab in patients with HER2-positive locally advanced breast cancer (NOAH): Follow-up of a randomised controlled superiority trial with a parallel HER2-negative cohort. Lancet Oncol..

[3] Pan H (2017). 20-year risks of breast-cancer recurrence after stopping endocrine therapy at 5 years. N. Engl. J. Med..

[4] Dar H (2021). Assessment of 25-year survival of women with estrogen receptor-positive/ERBB2-negative breast cancer treated with and without tamoxifen therapy: A secondary analysis of data from the stockholm tamoxifen randomized clinical trial. JAMA Netw. Open.

[5] Balic M, Thomssen C, Würstlein R, Gnant M, Harbeck NS, Gallen V (2019). St. Gallen/Vienna 2019: A brief summary of the consensus discussion on the optimal primary breast cancer treatment. Breast Care (Basel, Switzerland).

[6] Collins L (2011). Androgen receptor expression in breast cancer in relation to molecular phenotype: Results from the Nurses' Health Study. Mod. Pathol..

[7] McNamara K, Sasano H (2016). Androgen and breast cancer: An update. Curr. Opin. Endocrinol. Diabetes Obes..

[8] Jahan N, Jones C, Rahman R (2021). Androgen receptor expression in breast cancer: Implications on prognosis and treatment, a brief review. Mol. Cell. Endocrinol..

[9] De Amicis F (2010). Androgen receptor overexpression induces tamoxifen resistance in human breast cancer cells. Breast Cancer Res. Treat..

[10] Cochrane D (2014). Role of the androgen receptor in breast cancer and preclinical analysis of enzalutamide. Breast Cancer Res. BCR.

[11] Naderi A, Hughes-Davies L (2008). A functionally significant cross-talk between androgen receptor and ErbB2 pathways in estrogen receptor negative breast cancer. Neoplasia (New York, NY).

[12] Shi Y, Yang F, Huang D, Guan X (2018). Androgen blockade based clinical trials landscape in triple negative breast cancer. Biochim. Biophys. Acta. Rev. Cancer.

[13] Rebbeck T (1999). Modification of BRCA1-associated breast cancer risk by the polymorphic androgen-receptor CAG repeat. Am. J. Hum. Genet..

[14] Wang X, Zhang J, Liu X, Wei B, Zhan L (2021). Long noncoding RNAs in endometriosis: Biological functions, expressions, and mechanisms. J. Cell. Physiol..

[15] Schmidt K (2020). Targeting the oncogenic long non-coding RNA SLNCR1 by blocking its sequence-specific binding to the androgen receptor. Cell Rep..

[16] Yao M (2020). LINC00675 activates androgen receptor axis signaling pathway to promote castration-resistant prostate cancer progression. Cell Death Dis..

[17] Liu C, Wang X, Genchev G, Lu H (2017). Multi-omics facilitated variable selection in Cox-regression model for cancer prognosis prediction. Methods (San Diego, Calif.).

[18] Huang G, Zhou J, Chen J, Liu G (2022). Identification of pyroptosis related subtypes and tumor microenvironment infiltration characteristics in breast cancer. Sci. Rep..

[19] Kanehisa M, Furumichi M, Sato Y, Ishiguro-Watanabe M, Tanabe M (2021). KEGG: Integrating viruses and cellular organisms. Nucleic Acids Res..

[20] Subramanian A (2005). Gene set enrichment analysis: A knowledge-based approach for interpreting genome-wide expression profiles. Proc. Natl. Acad. Sci. U.S.A..

[21] Newman A (2015). Robust enumeration of cell subsets from tissue expression profiles. Nat. Methods.

[22] Bi F, Chen Y, Yang Q (2020). Significance of tumor mutation burden combined with immune infiltrates in the progression and prognosis of ovarian cancer. Cancer Cell Int..

[23] Chen W (2021). Identification of a tumor microenvironment-related gene signature indicative of disease prognosis and treatment response in colon cancer. Oxid. Med. Cell. Longev..

[24] Kramer I (2020). Breast cancer polygenic risk score and contralateral breast cancer risk. Am. J. Hum. Genet..

[25] Anestis A, Zoi I, Papavassiliou A, Karamouzis M (2020). Androgen receptor in breast cancer-clinical and preclinical research insights. Molecules (Basel, Switzerland)..

[26] Zhong H, Zeng G, He L (2021). Overexpression of the lncRNA AC012213.3 promotes proliferation, migration and invasion of breast cancer via RAD54B/PI3K/AKT axis and is associated with worse patient prognosis. Cancer Manag. Res..

[27] Nascimento G (2017). Molecular characterization of Koolen De Vries syndrome in two girls with idiopathic intellectual disability from central Brazil. Mol. Syndromol..

[28] Zhou W, Pang Y, Yao Y, Qiao H (2020). Development of a Ten-lncRNA signature prognostic model for breast cancer survival: A study with the TCGA database. Anal. Cell. Pathol. (Amst.).

[29] Yu Z (2021). Long non-coding RNA COL4A2-AS1 facilitates cell proliferation and glycolysis of colorectal cancer cells via miR-20b-5p/hypoxia inducible factor 1 alpha subunit axis. Bioengineered.

[30] Yao Y (2019). Integrated analysis of co-expression and ceRNA network identifies five lncRNAs as prognostic markers for breast cancer. J. Cell Mol. Med..

[31] Huang Z, Li J, Chen J, Chen D (2021). Construction of prognostic risk model of 5-methylcytosine-related long non-coding RNAs and evaluation of the characteristics of tumor-infiltrating immune cells in breast cancer. Front. Genet..

[32] Yu D, Wang X, Jin Z (2020). Linc00702 inhibits cell growth and metastasis through regulating PTEN in colorectal cancer. Eur. Rev. Med. Pharmacol. Sci..

[33] Li T (2019). LINC00702/miR-4652–3p/ZEB1 axis promotes the progression of malignant meningioma through activating Wnt/β-catenin pathway. Biomed. Pharmacother. Biomed. Pharmacother..

[34] Collina F (2019). HOTAIRLncRNA up-regulation is strongly related with lymph nodes metastasis and LAR subtype of Triple Negative Breast Cancer. J. Cancer.

[35] Yang F (2018). An androgen receptor negatively induced long non-coding RNA ARNILA binding to miR-204 promotes the invasion and metastasis of triple-negative breast cancer. Cell Death Differ..

[36] Dai H (2021). HIF1α regulates IL17 signaling pathway influencing sensitivity of taxane-based chemotherapy for breast cancer. Front. Cell Dev. Biol..

[37] Shuai C, Yang X, Pan H, Han W (2020). Estrogen receptor downregulates expression of PD-1/PD-L1 and infiltration of CD8 T cells by inhibiting IL-17 signaling transduction in breast cancer. Front. Oncol..

[38] Le T (2022). The ADAM9/UBN2/AKR1C3 axis promotes resistance to androgen-deprivation in prostate cancer. Am. J. Cancer Res..

[39] Bareche Y (2020). Unraveling triple-negative breast cancer tumor microenvironment heterogeneity: Towards an optimized treatment approach. J. Natl Cancer Inst..

[40] Yamaguchi M (2021). Androgens enhance the ability of intratumoral macrophages to promote breast cancer progression. Oncol. Rep..

