# Supplementary material for: Role of androgen receptor signaling pathway-related lncRNAs in the prognosis and immune infiltration of breast cancer
Source: Sci Rep. 2022 Nov 30;12:20631. doi: 10.1038/s41598-022-25231-0 (PMC9712677; doi:10.1038/s41598-022-25231-0)

**Figure S1. Survival analysis ARSP-lncrnas were closely associated with prognosis.** High expression of MAPT[–](https://www.so.com/link?m=bE6+HcXXZYcfI2XbEshJ2klvW/9/kFtyzujvIFG45rSTYqiGPJ1wXSsqmTgxCmxHSBOXc7IlzXfT/fhNM4HSWNwoUIiceNhSjx6hF9u4Gdm805SH9PpN6TpycSA4OkE2uN2ztJ8p+65mxvlPHWXcS/g==)IT1, AP005131.2, COL4A2[–](https://www.so.com/link?m=bE6+HcXXZYcfI2XbEshJ2klvW/9/kFtyzujvIFG45rSTYqiGPJ1wXSsqmTgxCmxHSBOXc7IlzXfT/fhNM4HSWNwoUIiceNhSjx6hF9u4Gdm805SH9PpN6TpycSA4OkE2uN2ztJ8p+65mxvlPHWXcS/g==)AS1, and AL807757.2 and low expression of AC012213.3 in the risk model had a better prognosis in breast cancer. The expression of AL117329.1, AC068189.1, LINC00702, AL121672.3 in the risk model had no significantly.


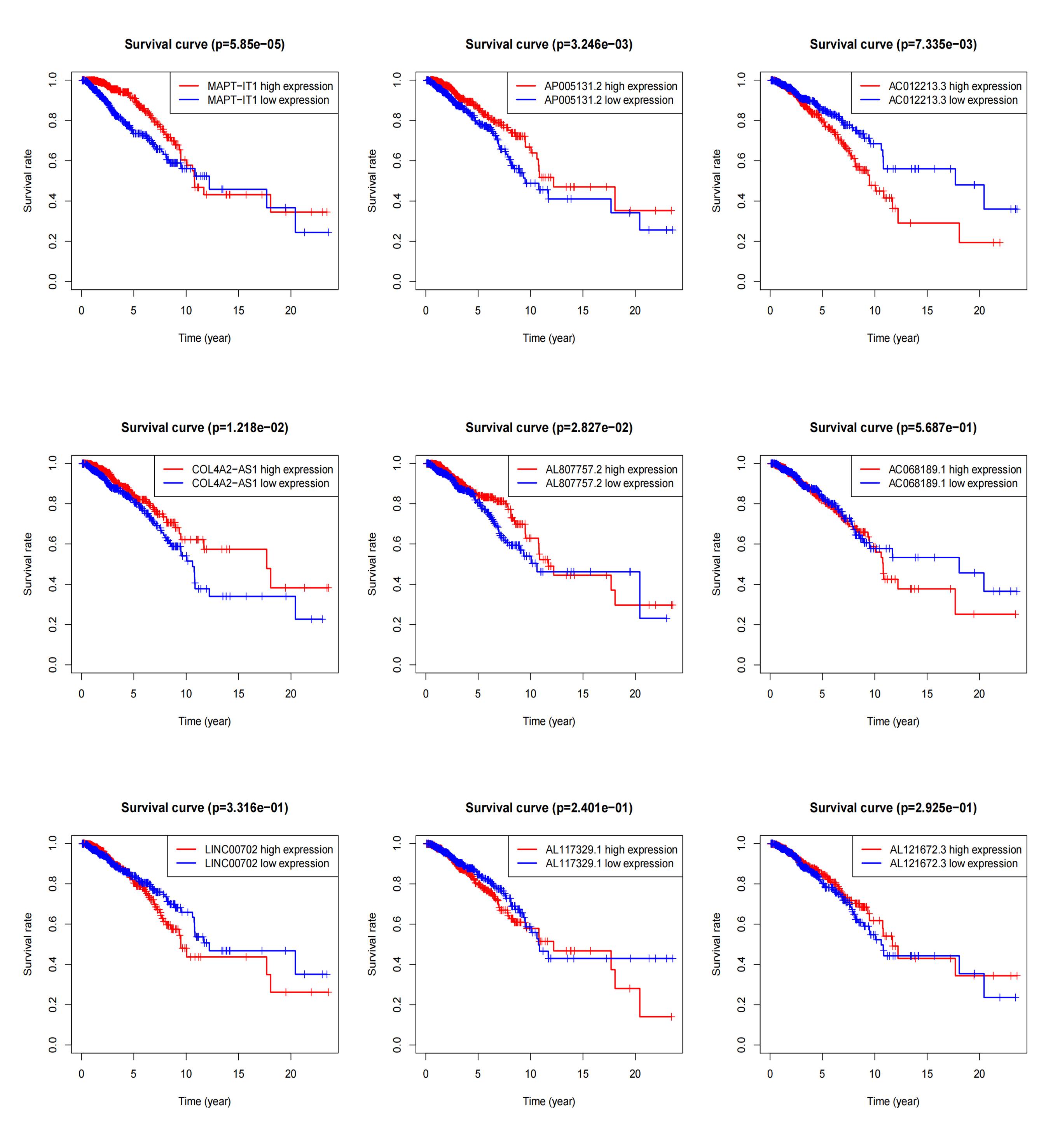


**Figure S2**. **Clinical significance of the androgen receptor signaling pathway-related lncRNAs score.** Kaplan–Meier survival curves of OS (survival probability) prognostic value stratifified by age, gender, T, N, M and stage between low- and high-risk groups in the total dateset.

**
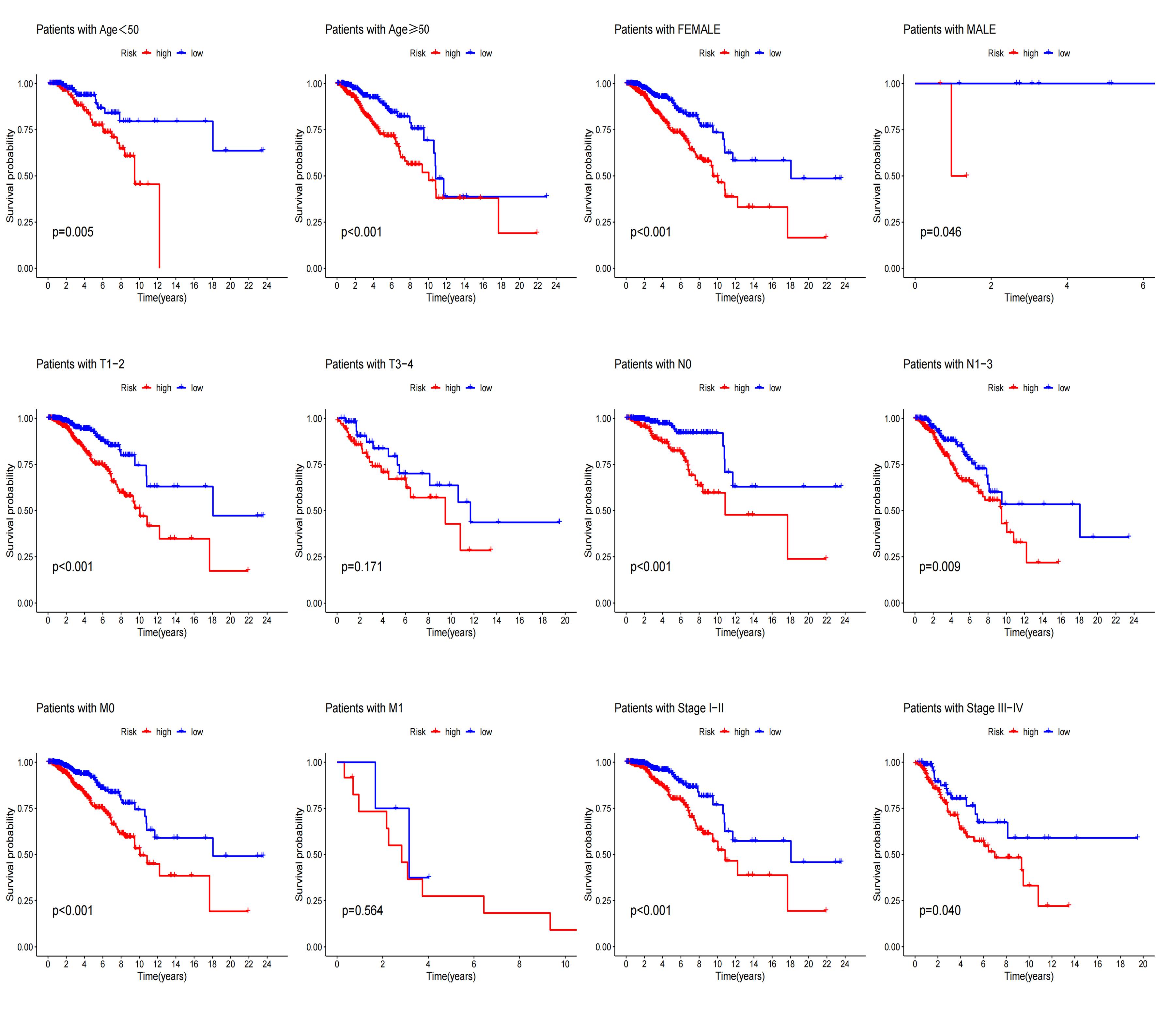
**

**Figure S3. Seven drugs solely showing signifificant IC50 difffference in risk model.** Nilotinib, Gefitinib, Epothilone. B, Elesclomol, Bosutinib, and Lenalidomide were lower in the low-risk group than the IC50 levels of these agents in the high-risk group. Bicalutamide was lower in the high-risk group than the IC50 of the same inhibitor in the low-risk group.

**
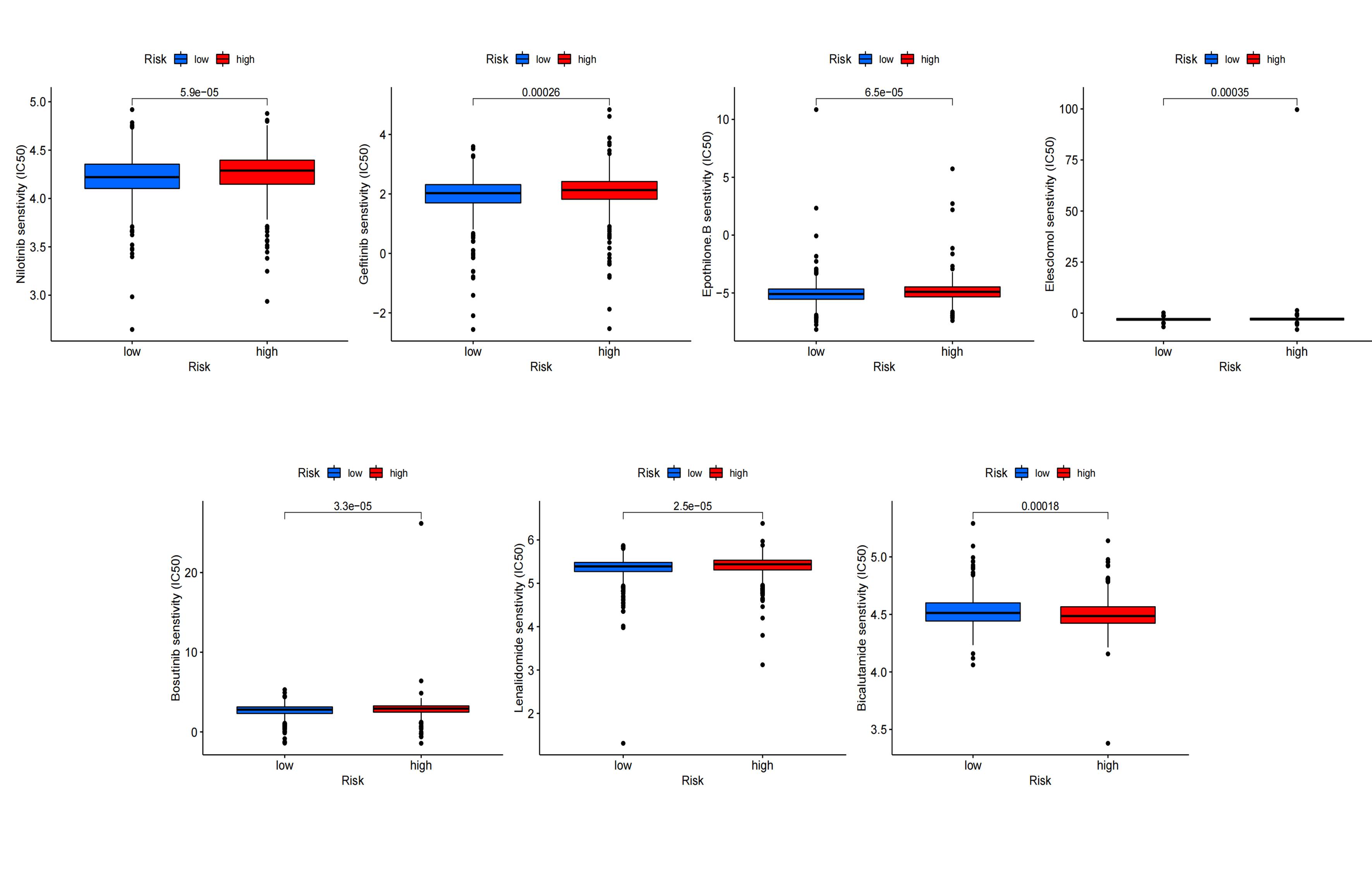
**

**Figure S4. Eleven drugs solely showing signifificant IC50 difffference in clusters. Docetaxel, Rapamycin, Sorafenib, Vinblastine and Salubrinal were** the maximum in Cluster C2, while the IC50 of Elesclomol and Nilotinib were the maximum in Cluster C1.


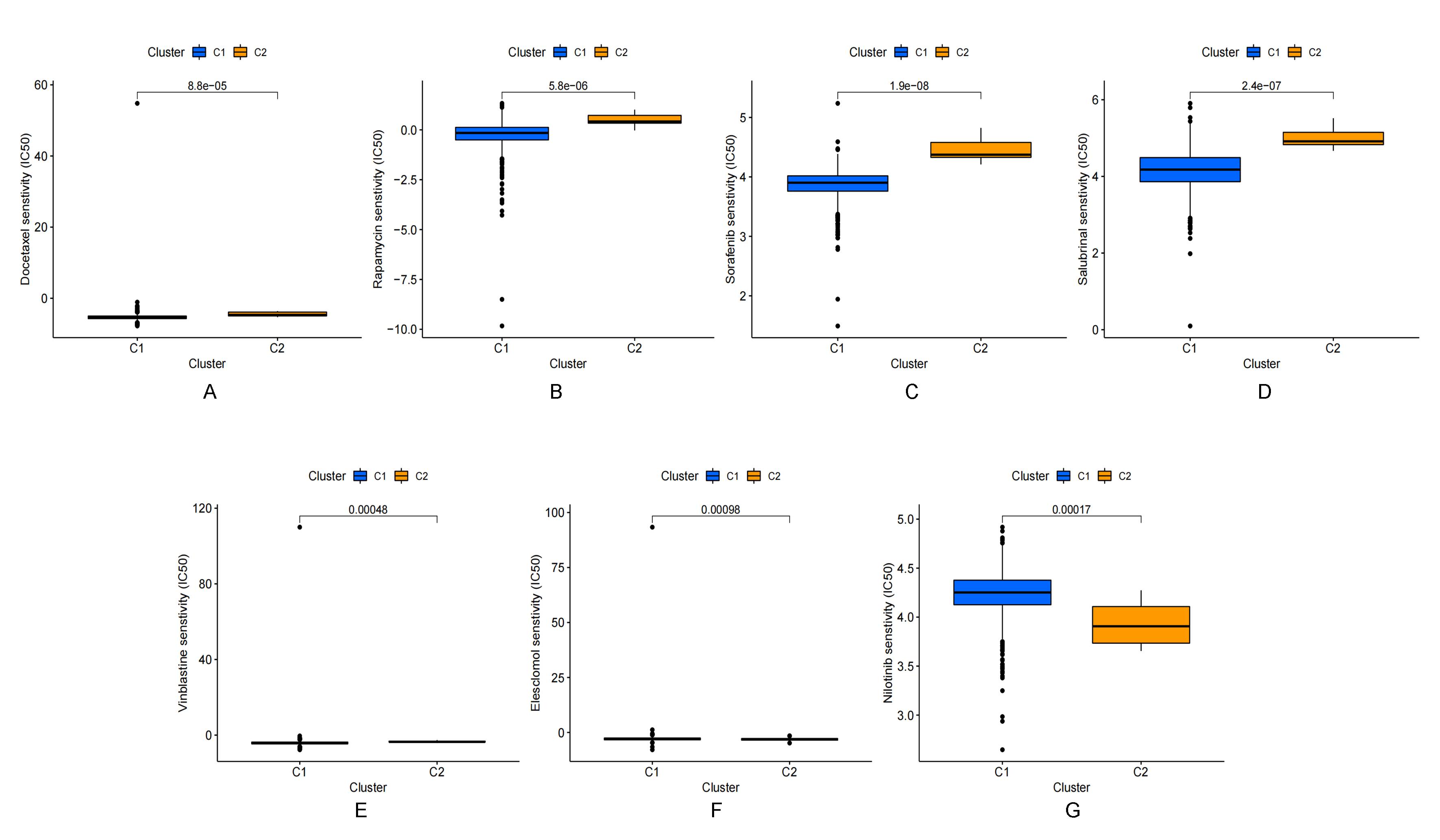

Supplement: Supplementary file 1 — Supplementary Figures. [file 41598_2022_25231_MOESM1_ESM.doc]
